# Trajectories of Energy Intake Distribution and Risk of Dyslipidemia: Findings from the China Health and Nutrition Survey (1991–2018)

**DOI:** 10.3390/nu13103488

**Published:** 2021-10-01

**Authors:** Xiaoyun Song, Huijun Wang, Chang Su, Zhihong Wang, Wenwen Du, Feifei Huang, Jiguo Zhang, Xiaofang Jia, Hongru Jiang, Yifei Ouyang, Li Li, Jing Bai, Xiaofan Zhang, Gangqiang Ding, Bing Zhang

**Affiliations:** Chinese Center for Disease Control and Prevention, National Institute for Nutrition and Health, Beijing 100050, China; sxydljk@126.com (X.S.); wanghj@ninh.chinacdc.cn (H.W.); suchang@ninh.chinacdc.cn (C.S.); wangzh@ninh.chinacdc.cn (Z.W.); Duww@ninh.chinacdc.cn (W.D.); huangff@ninh.chinacdc.cn (F.H.); zhangjg@ninh.chinacdc.cn (J.Z.); jiaxf@ninh.chinacdc.cn (X.J.); jianghr@ninh.chinacdc.cn (H.J.); ouyyf@ninh.chinacdc.cn (Y.O.); lili@ninh.chinacdc.cn (L.L.); Baijing@ninh.chinacdc.cn (J.B.); Zhangxf@ninh.chinacdc.cn (X.Z.); dinggq@chinacdc.cn (G.D.)

**Keywords:** energy intake, dyslipidemia, multi-trajectory model, cohort

## Abstract

Few studies have examined the secular trend of energy intake distribution. This study aims to describe trajectories of energy intake distribution and determine their association with dyslipidemia risk. Data of 2843 adult participants from the China Health and Nutrition Survey (CHNS) were analyzed. Trajectory groups of energy intake distribution were identified by multi-trajectory model over 27 years. Multilevel mixed-effects modified Poisson regression with robust estimation of variance was used to calculate risk ratio for incident dyslipidemia in a 9-year follow-up. Four trajectory groups were identified: “Energy evenly distributed group” (Group 1), “Lunch and dinner energy dominant group” (Group 2), “Dinner energy dominant group” (Group 3), “breakfast and dinner energy dominant group” (Group 4). Compared with Group 1, Group 3 was associated with higher risk of dyslipidemia (RR = 1.48, 95% CI = 1.26, 1.75), hypercholesterolemia (RR = 1.96, 95% CI = 1.37, 2.81) and high low-density lipoproteins cholesterols (LDL-C) (RR = 2.41, 95% CI = 1.82, 3.20). A U-shape was observed between cumulative average proportion of dinner energy and dyslipidemia risk (*p* for non-linear = 0.01), with stronger relationship at 40% and above. Energy intake distribution characterized by higher proportion of dinner energy, especially over 40% was associated with higher dyslipidemia risk in Chinese adults.

## 1. Introduction

Dyslipidemia is an important risk factor for cardiovascular disease (CVD). Evidences from clinical, genetic, and epidemiologic cohort studies consistently demonstrated that low-density lipoproteins cholesterols (LDL-C) causes atherosclerotic cardiovascular disease [1]. Meta-analysis of prospective studies reported positive association between elevated blood triglyceride (TG), total cholesterol (TC) and higher risk of cardiovascular diseases and all-cause mortality [2,3], while inverse associations of higher high-density lipoproteins cholesterols (HDL-C) and coronary heart disease or ischemic stroke were indicated by meta-analysis of prospective studies [4]. Dyslipidemia is closely related to diet and lifestyle. Modification of diet is one of the basic measures for the prevention and treatment of dyslipidemia [5]. A wealth of evidence has demonstrated the inverse association between lipid level and some food groups (for example fish, fruits, whole-grains), or specific dietary patterns (for example the MedDiet and the DASH diet) [6]. Besides, a growing body of studies suggests close relationships of eating patterns with metabolisms of lipid in both animals and humans. Rats fed in a delayed meal timing protocol increased the hepatic lipids and adipose tissue weight [7]. In general population, night-eating habit [8], taking large portion of energy in the later of the day [9,10], and having meals irregularly [11,12] were found to be positively associated with adverse cardiometabolic risk profiles.

Currently, literature on time-of-day of energy distribution and its relation with dyslipidemia is sparse. One study cross-sectionally examined the association between energy intake at different times of the day and blood lipid levels, but a full spectrum of energy distribution pattern across the day was not investigated in this study [10]. In contrast, other cross-sectional studies [13,14,15] have assessed daily energy distribution pattern in other population, however, the effect on dyslipidemia was not examined. Besides, to our knowledge, few studies has examined the secular trend of energy intake distribution, and no study has examined the longitudinal association between long-term energy intake distribution pattern and risk of dyslipidemia. Therefore, the aims of our study were to identify different trajectories of daily energy intake distribution, and to examine their longitudinal association with risks of dyslipidemia.

## 2. Materials and Methods

### 2.1. Study Design

The China Health and Nutrition Survey (CHNS) is a population-based longitudinal survey in China, ongoing since 1989. Follow-up surveys carried out in 1991, 1993, 1997, 2000, 2004, 2006, 2009, 2011, 2015, and 2018 collected dietary, anthropometric, clinical, and all other individual as well as household and community data. More details regarding the CHNS are provided in the previous article [16].

Our study included nine waves of survey data (CHNS 1991, 1993, 1997, 2000, 2004, 2006, 2009, 2015, and 2018). Because fasting blood lipids were only collected in waves 2009, 2015, and 2018, our cohort analysis for the association between trajectories of energy intake distribution and incident dyslipidemia was during waves of 2009, 2015, and 2018. To better represent long-term secular trend of energy intake distribution, energy intake proportions at three main meals from wave 1991 until the wave when participant was diagnosed with dyslipidemia were used to identify trajectories of daily energy intake distribution.

### 2.2. Study Participants

Participants aged ≥ 18 years, not during pregnant or lactating period, who had complete dietary data and reasonable daily energy intake (≥500 kcal and ≤5000 kcal) from 1991 to 2018, and had complete fasting blood lipid measurements from 2009 to 2018 were eligible for inclusion (*n* = 29,719). Among them, participants with at least 2 waves follow-ups from 2009 to 2018 and at least 3 waves of visits before 2009 were selected (*n* = 4289). Then, participants who were diagnosed with dyslipidemia when they first entered the cohort since 2009 were excluded (*n* = 1446). The final analytical sample included 2483 participants. The main reason for drop-out of participants was moving out of the original community with the process of urbanization. Table S1 in Supplemental Materials compares characteristics between included and excluded participants.

### 2.3. Calculation of Proportions of Energy Intake from Breakfast, Lunch and Dinner

In the CHNS, dietary data were collected based on a combination of three consecutive 24 h recalls (two weekdays and 1 weekend) at the individual level for food and beverages and a food inventory at the household level for condiments during the same three-day period. Information on types and amounts of food consumed at each meal during the previous 24 h were recorded by well-trained field interviewers. In our study, energy intake from both food and condiments at each meal was calculated by the China Food Composition. The proportions of energy intake at breakfast, lunch and dinner were firstly calculated for each recall day, then proportions of energy intake at breakfast, lunch and dinner were averaged across their consumption days to obtain mean estimates, respectively.

### 2.4. Outcome Measures

Overnight fasting blood samples were collected by trained nurses and biochemical indexes were measured in a national lab in Beijing with strict quality control. Plasma total triglycerides (TG), total cholesterol (TC), high-density lipoprotein cholesterol (HDL-C) and low-density lipoprotein cholesterol (LDL-C) were measured by CHOD-PAP (Kyowa Medex Co., Ltd., Tokyo, Japan) method.

Dyslipidemia was defined as TC ≥ 6.2 mmol/L (240 mg/dL) and/or TG ≥ 2.3 mmol/mol (200 mg/dL) and/or HDL-C < 1.0 mmol/L (40 mg/dL) and/or LDL-C ≥ 4.1 mmol/L (160 mg/dL), according to the 2016 Chinese guideline for the management of dyslipidemia in adults [17]. Besides, hypercholesterolemia was defined as TC ≥ 6.2 mmol/L (240 mg/dL); Hypertriglyceridemia was defined as TG ≥ 2.3 mmol/mol (200 mg/dL); Low HDL-C was defined as HDL-C < 1.0 mmol/L (40 mg/dL); High LDL-C was defined as LDL-C ≥ 4.1 mmol/L (160 mg/dL) [17].

### 2.5. Assessment of Covariates

We only assessed covariates in round 2009, 2015, and 2018, and both baseline and follow-up covariates were used in the present study. The following measures were considered covariates: age; sex; educational level (low (i.e., completed primary school), medium (i.e., completed middle school), high (i.e., completed high school and above); geographic region (urban and rural); total physical activity calculated into a metabolic equivalent of task (METs h/week) based on the Compendium of Physical Activities [18] (high, medium, and low, according to International physical activity questionnaire [19]); sleep duration (<6 h, 6–9 h, and >9 h); smoking (non-smoker and current smoker); alcohol drinking (non-drinker and current drinker); annual per capita household income; community urbanicity index, calculated based on 12 multidimensional components including physical, social, cultural and economic environment of the community [20]; chronic disease history (yes [i.e., ever diagnosed with hypertension or myocardial infarction or diabetes or apoplexy or cancer] no); total energy intake; CDGI (2019)-A score, calculated based on 13 food-related components and 1 nutrient-related component reflecting compliance for meeting the Chinese Dietary Guidelines 2016 [21]; body mass index (BMI); waist circumference (WC); systolic blood pressure (SBP); and diastolic blood pressure (DBP).

### 2.6. Statistical Analysis

We used a group-based multi-trajectory model [22] to identify trajectory groups of daily energy intake distribution based on proportions of energy intake from breakfast, lunch, and dinner. This approach allowed us to represent the longitudinal course of energy intake of three main meals jointly and to examine the association of secular trend of energy intake distribution and the subsequent risk of incident dyslipidemia. Models with 1 to 5 trajectory groups using censored normal distribution were fit respectively. We did not go beyond 5 groups for the sake of parsimony. Within each given number of trajectory groups, the polynomial orders (linear, quadratic, cubic, quartic, and quintic specifications) were tested for each trajectory shape until the best fitting model was established. Finally, the optimal number of trajectory groups was determined based on model-adequacy criteria [23] including the logged Bayes factor (≈2ΔBIC, >10), average posterior probability of assignment (APPA, >0.70), odds of correct classification (OCC, >5 for all groups), and proportion of individuals estimated to be assigned to each group (≥1% for each group).

Subsequent to model-selection, extracted trajectory groups were compared with respect to baseline demographic, lifestyle, and anthropometric variables with analysis of variance, Kruskal–Wallis and chi-square tests were used, where appropriate.

To evaluate the association between trajectory groups of energy intake distribution and risk of dyslipidemia, modified Poisson regression with robust (sandwich) estimation of variance was used, which is a reliable approach to estimate relative risk directly with binary outcomes [24]. Specifically, a three-level mixed-effects modified Poisson regression model with robust (sandwich) estimation of variance was used, taking household as the third level, individual as the second level, and repeated measurements of individual as the first level. Besides, subtypes of dyslipidemia were examined for their association with trajectory groups of energy intake distribution.

In the additional analysis, the three-level mixed-effects modified Poisson regression model with robust (sandwich) estimation of variance was used to investigate the association between quartiles of cumulative average of proportions of energy from breakfast, lunch, and dinner and risk of dyslipidemia, respectively. The cumulative average of proportions [25] of energy from breakfast, lunch, and dinner of a participant were calculated based on valid dietary assessments from the participant’s first entry into cohort since 1991 until the wave he/she was diagnosed with dyslipidemia, to better represent long-term eating behavior. Furthermore, the possible exposure–response relationships between proportions of energy from breakfast, lunch, and dinner and risk of dyslipidemia were explored, using a restricted cubic spline function with 4 knots (located at the 5th, 35th, 65th, and 95th percentiles). The 5th percentile was chosen to be the reference group for all spline plots (17.34% for breakfast, 26.09% for lunch, and 28.77% for dinner).

For all analysis, four models were fitted: Model 1 adjusted for no covariates. Model 2 adjusted for age, sex, marriage status, an education level, geographic region, annual per capita household income, urbanicity index, physical activity, smoking, alcohol drinking, sleep duration, and chronic disease history. Model 3 additionally adjusted for total energy intake and CDGI (2019)-A score. Model 4 additionally adjusted for BMI, WC, SBP, and DBP.

To assess the robustness of our main findings, we did two sensitivity analyses by restricting the analysis to participants without chronic disease history (hypertension or myocardial infarction or diabetes or apoplexy or cancer) to reduce the possibility of reverse causation bias caused by change of eating behavior when diagnosed with any chronic disease.

All the analyses were conducted in SAS 9.4 (SAS Institute, Inc., Cary, NC, USA) and Stata 15SE (Stata Corp., College Station, TX, USA). Group-based multi-trajectory model was conducted by package TRAJ for Stata [22]. *p* < 0.05 was considered statistically significant.

## 3. Results

### 3.1. Trajectory Groups of Energy Intake Distribution

**Figure 1:**
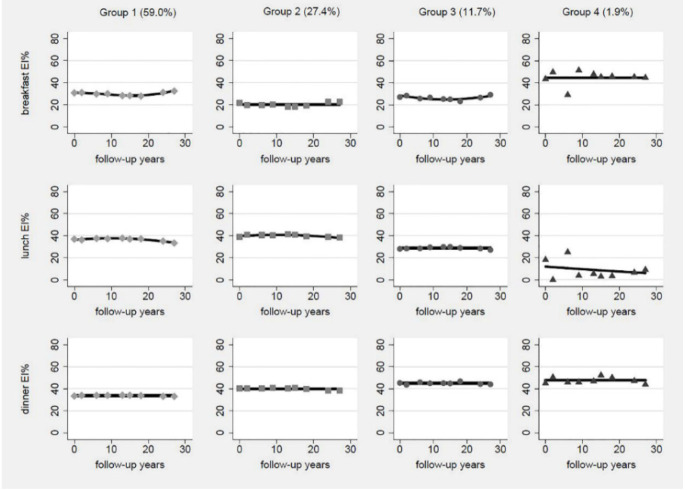
Estimated trajectory groups of energy intake distribution among Chinese adults. EI%, proportion of energy intake.

According to model-adequacy criteria, the goal of parsimony, and the rule of interpretability, we chose the 4-group solution (Figure 1). Table S2 in Supplemental Materials presents parameters of model-adequacy criteria.

Most trajectories of proportion of energy of breakfast, lunch, and dinner were relatively stable across the 27 years of follow-up period. The first group comprised 59.0% of the participants, characterized by about 30% energy intake (EI) from breakfast, 40% EI from lunch, and 30% EI from dinner. Thus, this group was labelled “energy evenly distributed group”. The second trajectory group comprised 27.4% of the participants, characterized by about 20% EI from breakfast, about 40% EI from lunch and 40% EI from dinner. So, this group was labelled “lunch and dinner energy dominant group”. The third trajectory group comprised 11.7% of the participants, characterized by about 25% EI from breakfast, 30% EI from lunch, and about 45% from dinner. Therefore, this group was labelled “dinner energy dominant group”. The fourth trajectory group comprised 1.9% of the participants, characterized by about 40% EI from breakfast, 15% EI from lunch with a downward trend, and 45% EI from dinner. Therefore, this group was labelled “breakfast and dinner energy dominant group”.

### 3.2. Baseline Characteristics by Trajectory Groups

In the baseline of follow-up between 2009 and 2018, participants in Group 1 were often women, married, had higher baseline mean level of BMI and DBP. Participants in Group 2 had higher education level, lower proportion of medium-to-high level of physical activity, higher level of urbanicity score, and higher mean level of WC. Participants in Group 3 had higher proportions of males and current drinker, with lower baseline mean level of BMI, WC, and SBP. Participants in Group 4 were older, had lower education level, lowest mean level of urbanicity score and highest baseline mean level of SBP (Table 1).

### 3.3. Trajectory Groups of Energy Intake Distribution and Dyslipidemia

Among the 2843 participants, the median follow-up time was 6 years, ranging from 3 years to 9 years. The total person-years and number of cases of dyslipidemia were 30,879 and 1073. Numbers of subtypes of outcome events for each trajectory group were presented in Table 2.

Associations between trajectory groups and risk of dyslipidemia are shown in Table 2. Group 1 was taken as the reference group, because previous studies suggested better cardiometabolic profiles associated with an energy balanced meal pattern [10,16,17]. In the longitudinal analysis, Group 3 were associated with higher risk of dyslipidemia (RR = 1.48, 95% CI = 1.26, 1.75), compared with Group 1. As for subtypes of dyslipidemia, Group 3 was associated with higher risk of hypercholesterolemia (RR = 1.96, 95% CI = 1.37, 2.81) and High LDL-C (RR = 2.41, 95% CI = 1.82, 3.20), compared with Group 1.

### 3.4. Cumulative Averages of Proportions of Energy from Breakfast, Lunch, Dinner and Dyslipidemia

Table 3 presents the association between cumulative averages of proportions of energy from breakfast, lunch, and dinner and risk of dyslipidemia. After adjusting for covariates, the RR (95% CI) for dyslipidemia was 0.82 (0.68, 0.98) for breakfast, 1.00 (0.84, 1.18) for lunch, and 1.35 (1.15, 1.59) for dinner, when comparing the highest with the lowest quartile.

**Figure 2:**
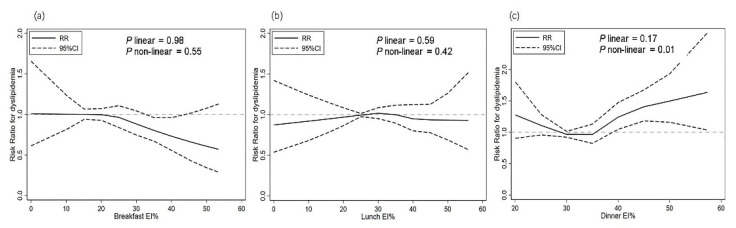
Exposure-response relationships between energy intake proportion at main meals and risk of dyslipidemia by restricted cubic spline for adjusted modified Poisson regression model with robust (sandwich) estimation of variance; EI%, energy intake proportion; (**a**) breakfast energy intake proportion and risk for dyslipidemia; (**b**) lunch energy intake proportion and risk for dyslipidemia; (**c**) dinner energy intake proportion and risk for dyslipidemia.

Figure 2 presents exposure–response relationships between energy intake proportion at meals and risk of dyslipidemia. The overall association between energy intake proportion of breakfast and risk of dyslipidemia was inverse and almost linear, but not statistically significant (*p* for linear = 0.64, *p* for non-linear = 0.96). No linear or non-linear relation were observed for association between energy intake proportion of lunch and risk of dyslipidemia (*p* for linear = 0.63, *p* for non-linear = 0.23). A significant non-linear relation was observed between energy intake proportion of dinner and risk of dyslipidemia (*p* for non-linear = 0.01), with stronger relationship at 40% and above.

### 3.5. Sensitivity Analysis

Figure S2 and Table S3 in Supplemental Materials present detailed results for the first sensitivity analysis. After restricting participants without chronic diseases, the identified trajectories were similar to those in the main analyses, and the subsequent risks of dyslipidemia by trajectory groups also yielded similar results as the main analyses.

## 4. Discussion

In this cohort of Chinese adults, we identified four distinct trajectory groups of energy intake distribution over 27 years, from 1991 to 2018. All patterns of energy intake distribution were found to be relatively stable over years. Trajectory group characterized by a stable “Dinner dominant” energy distribution was prospectively associated with higher risk of dyslipidemia, when compared with group characterized by a stable “evenly distributed” energy distribution. Moreover, when we examined the cumulative average proportion of single meal on a continuous scale, a significant nonlinear association between dinner energy proportion and dyslipidemia was found, with significantly higher risk at 40% and above.

There were limited data on secular trends in energy intake distribution, and their association with dyslipidemia, an important risk factor for CVD, have not been prospectively examined in free-living population. Almoosawi, et al. [26] described 17-year changes in energy and macronutrient intake across eating occasions in the 1946 British birth cohort. They found energy intake proportion has shifted towards later in the day in British adults between 1982 and 1999. In Almoosawi’s study, proportions of energy at different eating occasions were calculated as the mean value of the whole participants and examined separately, where heterogeneity in energy intake distribution could not be fully captured. Therefore, we used a group-based multi-trajectory model to identify heterogeneity of energy intake distribution in the population, and to consider the trajectories of three main meals jointly.

From 1991 to 2018, China witnessed a rapid change in eating environment and dietary pattern [27,28]. However, using group-based multi-trajectory model based on breakfast, lunch, and dinner energy proportion, we found the patterns of energy intake distribution of main meals were relatively stable across the 27 years of follow-up period in Chinese adults. Socio-economic factors influence eating behavior. Previous study [29] found dynamic shifts of increasing frequency of snacking occasions and energy proportion of snack with the economic growth between 1991 and 2009 in China. The relatively stable trajectories of energy intake distribution of main meals might be attributed to social-cultural habits and individual convenience. Further studies are needed to determine these factors.

We found four distinct energy intake distribution trajectory groups, of which “Energy evenly distributed group” (Group 1) including 59.0% of participants, “Lunch and dinner energy dominant group” (Group 2) including 27.4% of participants, “Dinner energy dominant group” (Group 3) including 11.7% of participants, and “breakfast and dinner energy dominant group” (Group 4) including 1.9% of participants, indicating most people kept a balanced meal pattern in our study. Although keeping a balanced meal pattern was indicated to be associated with lower BMI and SBP in previous researches [13,15,30], there are still scarce evidence as for which pattern of energy distribution is more beneficial or detrimental to health [31].

Few studies have investigated the associations between long-term of energy intake distribution and the risk of dyslipidemia in free-living population. Some previous researches [14,15,30] applied data-driven analytic approaches such as latent class analysis and cluster analysis to identify distinct and unknown patterns of energy intake distribution in the population, and cross-sectionally examined their relationships with obesity and hypertension. In another cross-sectional study, Chen et al. [10] studied the association between energy intake at different times of the day and blood lipid levels in an adult population in Taiwan. They found transferring 100 kcal intake at night (20:30–04:59) to the morning (05:00–09:29) or noon (11:30–13:29) would lower LDL-C by 46% and 27%, respectively. A similar association pattern was found for total cholesterol. In a cohort study in Japan adults [32], men and women with baseline night eating habits had greater risk of developing hypertriglyceridemia after an average of 3.9 follow-up years. A randomized crossover trial showed consuming a late dinner after 22:00 reduced fatty acid oxidation and mobilization [33]. Another randomized crossover trial showed that nighttime snacking increased total and LDL cholesterol and reduced fat oxidation [34]. Similar to these findings, our results suggested participants who constantly followed an eating pattern with higher proportion of energy intake later in the day had higher risk of developing dyslipidemia in an average of 6 years of follow-up. Complementarily, we added to the previous findings from the perspective of energy intake distribution, rather than isolated eating occasions, which could provide practical information in dietary intervention or guidance. What is more, we used trajectories of energy intake proportions, rather than single timepoint assessment, to better represent long-term effect of energy intake distribution on dyslipidemia.

In respect of subtype of dyslipidemia, higher risk of hypercholesterolemia and high LDL-C were associated with Group 3, which was similar with the findings of Chen’s study [35]. Plasma cholesterol level is regulated by the interplay between endogenous cholesterol synthesis, intestinal cholesterol absorption, and bile acid synthesis and excretion. Based on earlier studies, the endogenous cholesterol synthesis has a diurnal rhythm in human, with lowest rate during the day and highest during the night [36,37]. Besides, bile acid synthesis rate is lowest during the night and highest during the day [38]. If more energy is taken at the later time of day when cholesterol tends to be produced, circulating plasma cholesterol level would be elevated. As for LDL-C, study showed that human lipoprotein lipase activity was lower in the evening than in the morning [39]. Lipoprotein lipase activity was positively related with insulin sensitivity [40]. Since the diurnal rhythm of insulin sensitivity has been recognized, it is possible that decrease in insulin sensitivity leads to lower lipoprotein lipase, which results in higher circulating plasma LDL-C level in the evening if more energy was consumed.

Given the stable trend of trajectories found in our study, cumulative average values of proportion of energy from three main meals were calculated and examined their relationships with subsequent risk of dyslipidemia separately. Possible exposure–effect relationship was also investigated, which was rarely seen in previous studies. Results showed that a significant association was found between higher energy proportion of dinner and greater risk of dyslipidemia after adjusting for multiple covariates. When taken on a continuous scale, a U shape relationship was found for energy proportion of dinner and risk of dyslipidemia, where risk of dyslipidemia was lower at 30–35%, increased from 35% and reached statistical significance at 40%. Although a significant association was found between higher energy proportion of breakfast and lower risk of dyslipidemia, the effect size (0.82) was relatively small for a protective role and spline did not indicate statistical significance. These results suggested that higher proportion of energy of dinner might, to a larger extent, explain the increase of dyslipidemia in our study after adjusting for confounding factors. Besides, energy proportion of dinner of 40% might be the threshold of developing dyslipidemia in the adult population as indicated by our study, which needs further confirmation by more studies.

The strengths of our study include identifying secular trajectories of energy intake distribution by using long-term repeated data, long subsequent follow-up period, and inclusion of many related covariates. However, our study also has several limitations. Firstly, energy intake assessment was based on 3–24 h recalls, which was subject to recall bias. Secondly, although we adjusted for as many covariates as possible, the possibility of other confounders unable to be included in our study could not be ruled out. Thirdly, for the stability of trajectory model fitting and the exclusion of possible reverse causality bias, we carried out strict inclusion criteria for the study design, which might reduce the representativeness of the analytic sample and generalizability of the findings.

## 5. Conclusions

Four trajectory groups of energy intake distribution identified in the Chinese population from 1991 to 2018 were associated with different risks of dyslipidemia, with “Dinner energy dominant group” associated with increased subsequent risk of dyslipidemia, compared with “Energy evenly distributed group”. Over 40% of dinner energy proportion might be a threshold point for developing dyslipidemia. Future studies are warranted to unravel the biological pathways of these associations, and to demonstrate the effectiveness of adjusting energy intake distribution to prevent dyslipidemia.

## Figures and Tables

**Table 1:** Baseline characteristics by the four estimated latent trajectory groups.

Baseline Characteristics	Group 1	Group 2	Group 3	Group 4	*p* Value
	(*n* = 1676)	(*n* = 779)	(*n* = 334)	(*n* = 54)	
Age (year, mean [SD])	53.63 (11.55)	53.59 (12.38)	52.36 (11.50)	57.33 (9.25)	0.028
Sex (%)					
Male	720 (42.96)	376 (48.27)	164 (49.10)	26 (48.15)	0.027
Female	956 (57.04)	403 (51.73)	170 (50.90)	28 (51.85)	
Marriage status (%)					
In marriage	1531 (91.35)	710 (91.14)	293 (87.72)	49 (90.74)	0.212
Other status	145 (8.65)	69 (8.86)	41 (12.28)	5 (9.26)	
Education level (%)					
Primary school	817 (48.75)	330 (42.36)	165 (49.40)	39 (72.22)	<0.001
Middle school	573 (34.19)	283 (36.33)	130 (38.92)	12 (22.22)	
High school and above	286 (17.06)	166 (21.31)	39 (11.68)	3 (5.56)	
Geographic region (%)					
Urban	349 (20.82)	282 (36.20)	122 (36.53)	0 (0.00)	<0.001
Rural	1327 (79.18)	497 (63.80)	212 (63.47)	54 (100.00)	
Physical activity (%)					
Low	1480 (88.31)	725 (93.07)	291 (87.13)	47 (88.89)	0.004
Medium	127 (7.58)	39 (5.01)	34 (10.18)	4 (7.41)	
High	69 (4.12)	15 (1.93)	9 (2.69)	2 (3.70)	
Sleep duration (%)					
6~9 h	1467 (87.53)	676 (86.78)	300 (89.82)	49 (90.74)	0.341
<6 h	40 (2.39)	18 (2.31)	6 (1.80)	3 (5.56)	
>9 h	169 (10.08)	85 (10.91)	28 (8.38)	2 (3.70)	
Smoking (%)					
Nonsmoker	1240 (73.99)	554 (71.12)	231 (69.16)	38 (70.37)	0.273
Current smoker	436 (26.01)	225 (28.88)	103 (30.84)	16 (29.63)	
Alcohol drinking (%)					
Nondrinker	1151 (68.68)	511 (65.60)	206 (61.68)	41 (75.93)	0.029
Current drinker	525 (31.32)	268 (34.40)	128 (38.32)	13 (24.07)	
Chronic disease history (%)					
Yes	1463 (87.29)	689 (88.45)	303 (90.72)	47 (87.04)	0.343
No	213 (12.71)	90 (11.55)	31 (9.28)	7 (12.96)	
Per capita household income (yuan/year, median [IQR])	22,007 (11,065–40,371)	24,382 (11,579–48,569)	21,198 (12,690–36,015)	24,055 (12,991–37,231)	0.058
Urbanicity score (mean [SD])	62.37 (18.08)	70.09 (18.00)	59.72 (18.14)	56.82 (9.73)	<0.001
BMI (mg/kg, mean [SD])	23.37 (3.29)	22.80 (3.34)	22.06 (3.16)	22.92 (2.84)	<0.001
WC (cm, mean [SD])	80.00 (9.67)	80.60 (9.58)	77.89 (9.61)	78.51 (10.01)	<0.001
SBP (mmHg, mean [SD])	125.71 (18.46)	123.00 (18.35)	123.98 (18.05)	131.00 (19.61)	0.025
DBP (mmHg, mean [SD])	81.44 (11.08)	79.73 (10.39)	79.91 (10.79)	75.28 (12.46)	<0.001
CDGI (mean [SD])	45.51 (11.70)	45.30 (10.12)	45.61 (10.05)	43.34 (9.19)	0.529
Total energy (kcal, mean [SD])	2362.90 (774.13)	2413.84 (780.73)	2278.93 (693.68)	2441.83 (878.01)	0.124

**Table 2:** Association between trajectory groups and risk of dyslipidemia (*n* = 2843) ^1^.

Trajectory Groups	*n*	Cumulative Number of Cases/Person-Year	Model 1	Model 2	Model 3	Model 4
			Risk Ratio (95% CI)	Risk Ratio (95% CI)	Risk Ratio (95% CI)	Risk Ratio (95% CI)
Dyslipidemia						
Group 1	1676	606/18,225	1	1	1	1
Group 2	779	298/8397	1.06 (0.93, 1.21)	1.02 (0.89, 1.16)	1.03 (0.90, 1.18)	1.12 (0.98, 1.29)
Group 3	334	152/3702	1.25 (1.06, 1.47) **	1.32 (1.11, 1.56) **	1.31 (1.11, 1.56) **	1.48 (1.26, 1.75) ***
Group 4	54	17/555	0.90 (0.55, 1.47)	0.93 (0.56, 1.54)	0.90 (0.54, 1.49)	0.99 (0.60, 1.63)
Hypercholesterolemia						
Group 1	1676	158/18,225	1	1	1	1
Group 2	779	72/8397	0.99 (0.73, 1.33)	1.04 (0.76, 1.41)	1.07 (0.78, 1.45)	1.13 (0.83, 1.54)
Group 3	334	51/3702	1.62 (1.15, 2.27) **	1.86 (1.30, 2.65) **	1.87 (1.31, 2.66) **	1.96 (1.37, 2.81) ***
Group 4	54	4/555	0.83 (0.31, 2.18)	0.82 (0.30, 2.18)	0.83 (0.31, 2.20)	0.87 (0.33, 2.31)
Hypertriglyceridemia						
Group 1	1676	239/18,225	1	1	1	1
Group 2	779	124/8397	1.12 (0.89, 1.40)	1.06 (0.84, 1.34)	1.09 (0.86, 1.37)	1.25 (0.99, 1.59)
Group 3	334	49/3702	1.02 (0.75, 1.39)	1.03 (0.75, 1.40)	1.02 (0.74, 1.39)	1.23 (0.90, 1.68)
Group 4	54	10/555	1.36 (0.71, 2.58)	1.48 (0.77, 2.86)	1.44 (0.75, 2.77)	1.72 (0.91, 3.26)
Low HDL-C						
Group 1	1676	254/18,225	1	1	1	1
Group 2	779	136/8397	1.15 (0.93, 1.43)	1.03 (0.84, 1.28)	1.04 (0.84, 1.28)	1.14 (0.92, 1.41)
Group 3	334	45/3702	0.88 (0.64, 1.21)	0.89 (0.66, 1.22)	0.88 (0.64, 1.20)	1.02 (0.74, 1.38)
Group 4	54	6/555	0.76 (0.35, 1.67)	0.80 (0.36, 1.79)	0.74 (0.33, 1.66)	0.83 (0.38, 1.83)
High LDL-C						
Group 1	1676	207/18,225	1	1	1	1
Group 2	779	97/8397	1.01 (0.78–1.30)	0.98 (0.75–1.28)	1.02 (0.78–1.32)	1.08 (0.83–1.41)
Group 3	334	80/3702	1.92 (1.47–2.51) ***	2.21 (1.67–2.92) ***	2.23 (1.68–2.95) ***	2.41 (1.82–3.20) ***
Group 4	54	5/255	0.78 (0.34–1.80)	0.77 (0.33–1.80)	0.79 (0.34–1.83)	0.83 (0.36–1.95)

^1^ A three-level mixed-effects Poisson regression with robust (sandwich) estimation of variance, taking household as the third level, individual as the second level, and repeated measurements of individual as the first level. Model 1 adjusted for no covariates. Model 2 adjusted for age, sex (categorical), marriage status (categorical), an education level (categorical), geographic region (categorical), per capita household income, urbanicity index, physical activity (categorical), smoking (categorical), alcohol drinking (categorical), sleep duration (categorical), and chronic disease history (categorical). Model 3 additionally adjusted for total energy intake and CDGI (2019)-A score. Model 4 additionally adjusted for BMI, WC, SBP, and DBP. ** *p* < 0.01, *** *p* < 0.001.

**Table 3:** Association between cumulative averages of proportions of energy from breakfast, lunch, and dinner and risk of dyslipidemia (*n* = 2843) ^1^.

		*n*	Cumulative Number of Cases/Person-Year	Model 1	Model 2	Model 3	Model 4
				Risk Ratio (95% CI)	Risk Ratio (95% CI)	Risk Ratio (95% CI)	Risk Ratio (95% CI)
Breakfast							
Q1	<22.9%	710	276/7761	1	1	1	1
Q2	22.9–26.9%	709	282/7839	1.01 (0.86, 1.18)	1.04 (0.88, 1.22)	1.04 (0.89, 1.22)	1.01 (0.86, 1.19)
Q3	26.9–31.0%	713	279/7797	1.01 (0.86, 1.18)	1.04 (0.88, 1.23)	1.03 (0.87, 1.21)	0.93 (0.79, 1.10)
Q4	≥31.0%	711	236/7482	0.86 (0.73, 1.02)	0.95 (0.79, 1.14)	0.91 (0.76, 1.09)	0.82 (0.68, 0.98) *
*p* trend				0.06	0.405	0.214	0.010
Lunch							
Q1	<33.1%	708	268/7761	1	1	1	1
Q2	33.1–36.5%	713	287/7839	1.07 (0.91, 1.25)	1.01 (0.85, 1.18)	1.01 (0.86, 1.19)	0.99 (0.84, 1.17)
Q3	36.5–39.7%	711	243/7797	0.91 (0.77, 1.07)	0.84 (0.71, 1.00)	0.87 (0.73, 1.03)	0.85 (0.71, 1.01)
Q4	≥39.7%	711	275/7482	1.03 (0.88, 1.20)	0.97 (0.82, 1.14)	1.01 (0.86, 1.19)	1.00 (0.84, 1.18)
*p* trend				0.841	0.38	0.743	0.598
Dinner							
Q1	<33.3%	710	258/7761	1	1	1	1
Q2	33.3–36.5%	711	238/7839	0.92 (0.78, 1.09)	0.92 (0.77, 1.09)	0.90 (0.75, 1.07)	0.91 (0.77, 1.08)
Q3	36.5–40.5%	708	268/7797	1.04 (0.88, 1.22)	1.00 (0.95, 1.18)	0.99 (0.83, 1.17)	1.07 (0.90, 1.27)
Q4	≥40.5%	714	309/7482	1.20 (1.02, 1.40) *	1.22 (1.04, 1.44) *	1.19 (1.01, 1.40) *	1.35 (1.15, 1.59) **
*p* trend				0.002	0.005	0.013	<0.001

^1^ A three-level mixed-effects Poisson regression with robust (sandwich) estimation of variance, taking household as the third level, individual as the second level, and repeated measurements of individual as the first level. Model 1 adjusted for no covariates. Model 2 adjusted for age, gender (categorical), marriage status (categorical), an education level (categorical), geographic region (categorical), per capita household income, urbanicity index, physical activity (categorical), smoking (categorical), alcohol drinking (categorical), sleep duration (categorical), and chronic disease history (categorical). Model 3 additionally adjusted for total energy intake and CDGI (2019)-A score. Model 4 additionally adjusted for BMI, WC, SBP, and DBP. * *p* < 0.05, ** *p* < 0.01. *p* trend was examined by assigning the median value of each quartile as a continuous variable.

## Data Availability

The datasets generated during and/or analyzed during the current study are available from the corresponding authors (B.Z.) on reasonable request.

## Supplementary Materials

The following are available online at https://www.mdpi.com/article/10.3390/nu13103488/s1, Supplemental Figure S1: Flow chart of study population selection, Supplemental Table S1: Comparison of characteristics between included and excluded participants, China Health and Nutrition Survey 1991–2018, Supplemental Table S2: Parameters of model-adequacy criteria of the multi-trajectory model, Supplemental Figure S2: Estimated trajectory groups of energy intake distribution among Chinese adults without chronic diseases (*n* = 2036), Supplemental Table S3: Association between trajectory groups and risk of dyslipidemia in Chinese adults without chronic diseases (*n* = 2036).
