# Supplementary material for: Trajectories of Energy Intake Distribution and Risk of Dyslipidemia: Findings from the China Health and Nutrition Survey (1991–2018)
Source: Nutrients. 2021 Oct 1;13(10):3488. doi: 10.3390/nu13103488 (PMC8538511; doi:10.3390/nu13103488)
Supplement: Supplementary file 1 [file nutrients-13-03488-s001.zip › Additional File 2 Supplemental Table S1.pdf]

Table S1. Comparison of baseline characteristics between included and excluded participants[1]

Baseline characteristics	Excluded(n=26876)	Included(n=2843)	P value
Age (year, median [IQR])	46.8(33.1,61.0)	53.6(44.8,61.8)	<0.001
Gender (%)			0.06
Man	47.1	45.2	
Woman	52.9	54.8	
Marriage status (%)			<0.001
In marriage	74.9	90.9	
Other status	25.1	9.2	
Geographic region (%)			<0.001
Urban	40.6	26.5	
Rural	59.4	73.5	
Smoking (%)			<0.001
Nonsmoker	75.5	72.6	
Current smoker	24.5	27.4	
Drinking (%)			0.90
nondrinker	67.3	67.2	
Current drinker	32.7	32.9	
Education level (%)			<0.001
Primary school	32.9	47.5	
Middle school	33.2	35.1	
High school and above	33.9	17.4	
Physical activity (%)			<0.001
Low	94.8	89.5	
Medium	3.7	7.2	
High	1.5	3.3	
Chronic disease history (%)			0.06
No	86.8	88.0	
Yes	13.2	12.0	
Per capita household income (yuan/year, median [IQR])	22169(7893,54168)	22299(11488,41426)	0.85
Urbanicity score (median [IQR])	69.6 (51.8,84.8)	59.5 (49.7,81.9)	<0.001
BMI (mg/kg^2, mean [SD])	23.4(3.7)	23.1 (3.3)	<0.001
WC (cm, mean [SD])	82.1(11.5)	81.6(9.9)	0.05
SBP (mmHg, mean [SD])	123.9(19.4)	124.9(18.5)	0.01
DBP (mmHg, mean [SD])	79.5(11.5)	80.5(11.3)	<0.001
TC (mmol/L, mean [SD])	4.9(1.1)	4.6(0.7)	<0.001
TG (mmol/L, median [IQR])	1.3(0.9,2.1)	1.1(0.8,1.4)	<0.001
HDL-C (mmol/L, mean [SD])	1.3(0.4)	1.5(0.4)	<0.001

LDL-C (mmol/L, mean [SD])	3.1(1.0)	2.9(0.6)	<0.001

[1] Comparison of characteristics in the baseline of follow-up between 2009 and 2018. If participants did not visit from 2009 onward, then the year closest to 2009 was taken as baseline.
