# Supplementary material for: Trajectories of Energy Intake Distribution and Risk of Dyslipidemia: Findings from the China Health and Nutrition Survey (1991–2018)
Source: Nutrients. 2021 Oct 1;13(10):3488. doi: 10.3390/nu13103488 (PMC8538511; doi:10.3390/nu13103488)
Supplement: Supplementary file 1 [file nutrients-13-03488-s001.zip › Additional File 3 Supplemental Table S2.pdf]

Table S2. Parameters of model-adequacy criteria of the multi-trajectory model

Trajectory group number	2ΔBIC	APPA	OCC	Entropy	Proportion of individuals in groups (%)
1	NA	NA	NA	1	100
2	4014.96	0.93	9.77/19.21	0.771	61.58/38.42
3	4456.44	0.94	11.60/19.92/4476.06	0.861	60.94/36.91/2.15
4	1577.94	0.93	11.37/21.96/75.24/5324.30	0.858	58.33/27.76/12.03/1.89
5	1018.96	0.91	12.37/20.84/85.83/6.40/7955.07	0.849	49.63/29.23//10.65/8.62/1.87

2ΔBIC, ≈the logged Bayes factor, > 10 was considered as a reasonable standard for strong evidence in favor of the complex model); APPA, average posterior probability of assignment, > 0.70 was indicative of a good model fit; OCC, odds of correct classification, > 5 for all groups was indicative of a good model fit; Entropy, >0.80 was indicative of better classification; Proportion of individuals in group, ≥ 1% for each group was accepted.
