# Supplementary material for: Trajectories of Energy Intake Distribution and Risk of Dyslipidemia: Findings from the China Health and Nutrition Survey (1991–2018)
Source: Nutrients. 2021 Oct 1;13(10):3488. doi: 10.3390/nu13103488 (PMC8538511; doi:10.3390/nu13103488)
Supplement: Supplementary file 1 [file nutrients-13-03488-s001.zip › Additional File 4 Supplemental Figure S2.pdf]

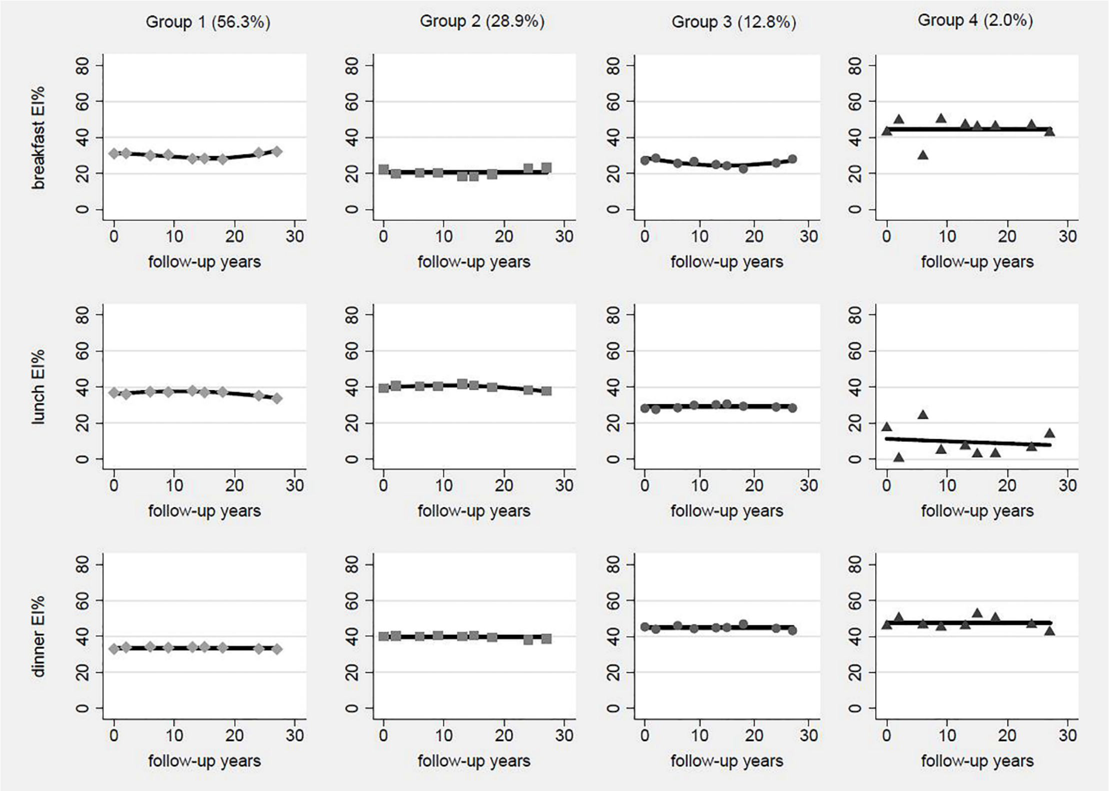

Figure S2. Estimated trajectory groups of energy intake distribution among Chinese adults without chronic diseases (n=2036)
