# Supplementary material for: Trajectories of Energy Intake Distribution and Risk of Dyslipidemia: Findings from the China Health and Nutrition Survey (1991–2018)
Source: Nutrients. 2021 Oct 1;13(10):3488. doi: 10.3390/nu13103488 (PMC8538511; doi:10.3390/nu13103488)
Supplement: Supplementary file 1 [file nutrients-13-03488-s001.zip › Additional File 5 Supplemental Table S3.pdf]

Table S3. Association between trajectory groups and risk of dyslipidemia in Chinese adults without chronic diseases (n=2036) [1]

Trajectory groups	N	Cumulative number of cases/person-year	Model 1	Model 2	Model 3	Model 4
			Risk ratio (95% CI)	Risk ratio (95% CI)	Risk ratio (95% CI)	Risk ratio (95% CI)
Dyslipidemia						
Group 1	1146	383/12252	1	1	1	1
Group 2	589	218/6213	1.11(0.95,1.31)	1.04(0.88,1.23)	1.05(0.89,1.24)	1.12(0.95,1.33)
Group 3	260	107/2898	1.21(0.99,1.48)	1.24(1.01,1.53) *	1.25(1.01,1.53) *	1.44(1.17,1.78) **
Group 4	41	15/405	1.15(0.69,1.93)	1.18(0.69,2.02)	1.17(0.69,1.99)	1.21(0.73,2.02)
Hypercholesterolemia						
Group 1	1146	105/12252	1	1	1	1
Group 2	589	53/6213	0.99(0.70,1.40)	1.00(0.70,1.44)	1.04(0.72.1.49)	1.10(0.76,1.58)
Group 3	260	34/2898	1.42(0.95,2.13)	1.57(1.04,2.37) *	1.58(1.05.2.39) *	1.65(1.08,2.50) *
Group 4	41	3/405	0.85(0.28,2.63)	0.84(0.27,2.65)	0.87(0.28.2.73)	0.90(0.28,2.83)
Hypertriglyceridemia						
Group 1	1146	147/12252	1	1	1	1
Group 2	589	88/6213	1.16(0.88,1.54)	1.05(0.79,1.41)	1.07(0.81.1.43)	1.19(0.89,1.61)
Group 3	260	36/2898	1.06(0.74,1.52)	1.03(0.72,1.48)	1.03(0.72.1.48)	1.12(0.91,1.90)
Group 4	41	10/405	2.01(1.07,3.75) *	2.23(1.17,4.23) *	2.20(1.17.4.15) *	2.36(1.24,4.50) **
Low HDL_C						
Group 1	1146	150/12252	1	1	1	1
Group 2	589	96/6213	1.03(0.77,1.39)	1.06(0.81,1.38)	1.07(0.82.1.39)	0.98(0.66,1.47)
Group 3	260	31/2898	1.82(1.33,2.49)	0.85(0.57,1.26)	0.84(0.57.1.25)	1.13(0.86,1.47)
Group 4	41	5/405	0.65(0.22,1.94)	1.08(0.46,2.57)	1.04(0.43.2.46)	1.05(0.45,2.45)
High LDL_C						
Group 1	1146	136/12252	1	1	1	1
Group 2	589	72/6213	1.25(0.96,1.62)	1.02(0.75,1.39)	1.06(0.78.1.44)	1.13(0.83,1.54)
Group 3	260	57/2898	0.89(0.60,1.33)	2.05(1.49,2.84) ***	2.08(1.50.2.87) ***	2.32(1.67,3.23) ***
Group 4	41	3/405	0.98(0.42,2.30)	0.62(0.21,1.87)	0.66(0.22.1.97)	0.67(0.23,1.95)

[1] A three-level mixed-effects Poisson regression with robust (sandwich) estimation of variance, taking household as the third level, individual as the second level, and repeated measurements of individual as the first level. Model 1 adjusted for no covariates. Model 2 adjusted for age, gender (categorical), marriage status(categorical), an education level (categorical), geographic region (categorical), per capita household income, urbanicity index, physical activity (categorical), smoking (categorical), alcohol drinking (categorical), sleep duration (categorical), and chronic disease history (categorical). Model 3 additionally adjusted for total energy intake and CDGI (2019)-A score. Model 4 additionally adjusted for BMI, WC, SBP, and DBP. * P < 0.05, ** P < 0.01, **P < 0.001.
