# Supplementary figures and images for: Trajectories of Energy Intake Distribution and Risk of Dyslipidemia: Findings from the China Health and Nutrition Survey (1991–2018)
Source: Nutrients. 2021 Oct 1;13(10):3488. doi: 10.3390/nu13103488 (PMC8538511; doi:10.3390/nu13103488)

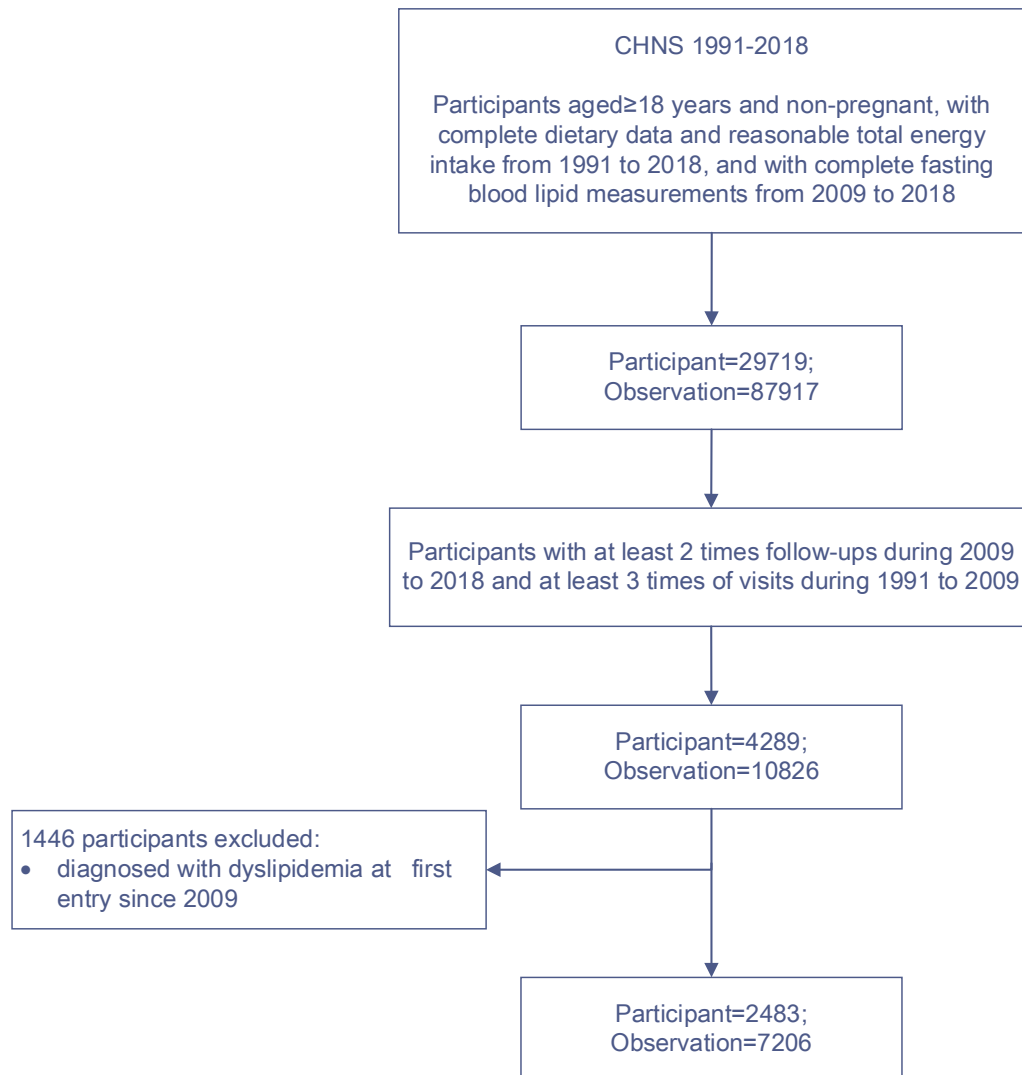

**Figure S1.** Flow chart of study population selection

Supplement: Supplementary file 1 [file nutrients-13-03488-s001.zip › Additional File 1 Supplemental Figure S1.pdf]
